# N6-Methyladenosine RNA Modification in the Tumor Immune Microenvironment: Novel Implications for Immunotherapy

**DOI:** 10.3389/fimmu.2021.773570

**Published:** 2021-12-09

**Authors:** Liting Guo, Hui Yang, Chenfei Zhou, Yan Shi, Lei Huang, Jun Zhang

**Affiliations:** Department of Oncology, Ruijin Hospital, Shanghai Jiao Tong University School of Medicine, Shanghai, China

**Keywords:** N6-methyladenosine methylation, m^6^A, tumor immunity, immunotherapy, tumor immune microenvironment

## Abstract

N6-methyladenosine (m^6^A) methylation is one of the most common modifications of RNA in eukaryotic cells, and is mainly regulated by m^6^A methyltransferases (writers), m^6^A demethylases (erasers), and m^6^A binding proteins (readers). Recently, accumulating evidence has shown that m^6^A methylation plays crucial roles in the regulation of the tumor immune microenvironment, greatly impacting the initiation, progression, and metastasis processes of various cancers. In this review we first briefly summarizes the m^6^A-related concepts and detection methods, and then describes in detail the associations of m^6^A methylation modification with various tumor immune components especially immune cells (e.g., regulatory T cells, dendritic cells, macrophages, and myeloid-derived suppressor cells) in a variety of cancers. We discuss the relationship between m^6^A methylation and cancer occurrence and development with the involvement of tumor immunity highlighted, suggesting novel markers and potential targets for molecular pathological diagnosis and immunotherapy of various cancers.

## Introduction

**Figure 1:**
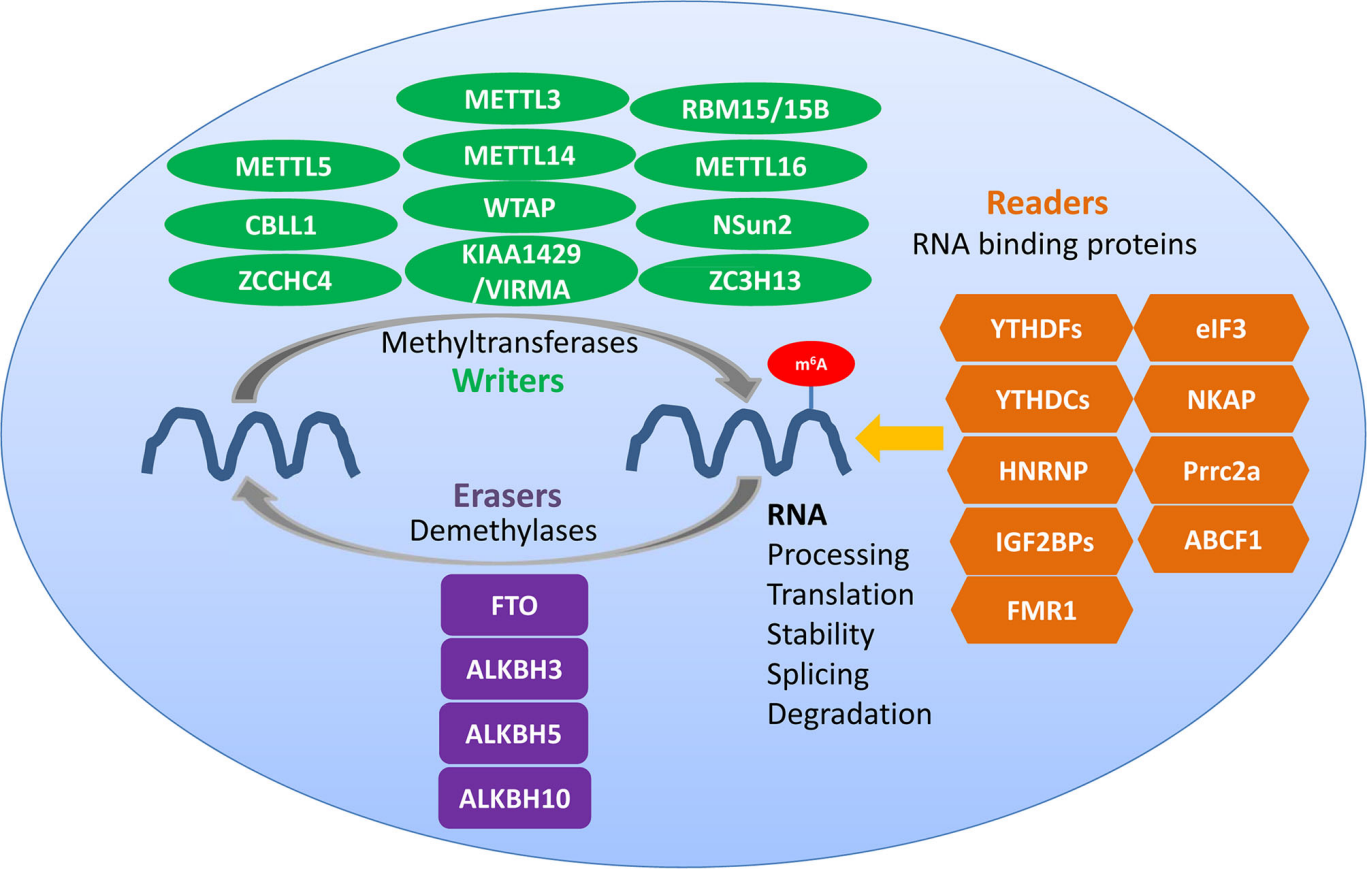
Regulators of m^6^A methylation within immune cells. Writers, erasers, and readers play different roles in the dynamic m^6^A modification of RNA. Common writers include METTL3, METTL14, and WTAP, common erasers include FTO and ALKBH5, and common readers include YTHDF1/2/3, YTHDC1/2, HNRNP, and IGF2BPs.

N6-methyladenosine (m^6^A) methylation is one of the most abundant RNA modifications in eukaryotic cells (1, 2). It involves three kinds of vital regulatory proteins, namely writers, erasers, and readers (2), which can respectively add, remove, and preferentially bind to the m^6^A modification sites and modulate the fate of RNA (1). m^6^A modification is a key regulator of diverse RNA biology processes (**Figure 1**), including RNA processing, translation, stabilization, splicing, and degradation (3, 4). Recently, accumulating evidence has revealed the potential links between m^6^A modification and cancer immunology (5, 6). m^6^A modification plays vital roles in diverse tumor immunity processes among a variety of cancers, affecting the development, proliferation, growth, invasion, and metastasis of cancers (5). The tumor microenvironment (TME) is the environment around tumor cells, including the surrounding blood vessels, immune cells, fibroblasts, molecules, the extracellular matrix, and other stromal components. In this review, we discuss the characteristics of m^6^A modulators and the immunomodulatory function of m^6^A methylation in the tumor immune microenvironment (TIME), which refers to the immune and immune-associated components of the TME, and which is a complex interactive network consisting of various immune cells, cytokines, and fibroblasts that plays important roles in tumor initiation, progression, metastasis, and treatment response (7, 8). We focus on the associations of m^6^A modification in the TIME with cancer immunity and immunotherapy.

## Regulation of m^6^A Methylation by m^6^A Writers, Erasers, and Readers

### m^6^A Writers

m^6^A writers are generally considered to be composed of m^6^A methylases, majorly including methyltransferase-like 3 (METTL3), methyltransferase-like 14 (METTL14), Wilms’ tumor 1-associating protein (WTAP), RNA binding motif protein 15 (RBM15) and its paralog RBM15B, which form the methyltransferase complex (MTC) (9). m^6^A writers are responsible for writing methylation information into RNA (6, 10). METTL3, a 70-kDa protein of the first identified m^6^A methyltransferases in eukaryotes, is a key enzymatic component of the MTC (6, 11); it can combine with S-adenosyl methionine (SAM) and transfer a methyl group to RNA. METTL4 is responsible for the recognition of substrates and functions as an allosteric activator that also binds to the target RNA (10–12). METTL14 serves as the RNA-binding platform, promoting the translation of related genes and enhancing the complex integrity (13, 14). METTL3 can form a heterodimer complex with the homologous protein METTL14. The METTL3-METTL14 dimer complex induces m^6^A deposition in transcripts on nuclear RNA (10). RBM15/15B interacts with METTL3 in a WTAP-dependent manner to help recruit the complexes to methylate-specific sites (15). Other m^6^A methyltransferases such as methyltransferase-like 16 (METTL16), zinc finger CCCH domain-containing protein 13 (ZC3H13), KIAA1429 [also known as vir-like m^6^A methyltransferase associated (VIRMA)], and NOP2/Sun domain family, member 2 (NSun2) are also essential for the formation of the MTC (16–21).

### m^6^A Erasers

Obesity-associated protein FTO and alkB homolog 5 (ALKBH5) are m^6^A demethylases which are also called erasers and which ensure that m^6^A modification is a dynamic and reversible process (22). The RNA m^6^A modification can be removed by the demethylases FTO and ALKBH5. FTO was the first protein discovered to catalyze m^6^A demethylation, and knocking down the expression of FTO can increase the m^6^A methylation level of RNA. In contrast, when FTO is overexpressed, the m^6^A level of intracellular RNA is suppressed (23). FTO is located in nuclear speckles, where the m^6^A MTC also locates, and can reverse the m^6^A modification of RNA. ALKBH5 was the second m^6^A demethylase identified that could oxidatively reverse m^6^A modifications (22, 24). It is a Fe^2+^- and α-ketoglutarate-dependent non-heme oxygenase that can oxidize the N-methyl group at the m^6^A methylation site to hydroxymethyl group. ALKBH5 is also mainly located in nuclear speckles, and depends on its demethylase activity to affect the transport of RNA out of the nucleus; it then further modulates nuclear RNA metabolism and gene expression (22–25).

### m^6^A Readers

The RNA-binding proteins that bind to m^6^A modification sites are called m^6^A readers, which include the YTH domain family (YTHDF1/2/3 and YTHDC1/2), heterogeneous nuclear ribonucleoproteins (HNRNPs; hnRNPC, hnRNPG, and hnRNPA2B1), and insulin-like growth factor 2 mRNA-binding proteins (IGF2BP1-3); they can specifically bind to the m^6^A methylation sites affecting RNA metabolism, and are responsible for reversing or eliminating the RNA modification (23, 26–31). YTHDF1 interacts with translation initiation factors to promote translation and to reduce the binding of ribosomes to m^6^A-modified RNA, which promotes the degradation of RNA. YTHDF2 accelerates the decay of m^6^A-methylated RNA, and YTHDF3 can promote the translation promoted by YTHDF1 and regulate the YTHDF2-mediated RNA-decay-promotion (13, 29). The recognition of the ribonucleoprotein HNRNPC/G and its binding to the m^6^A modification sites are also indirect, which are mediated by the m^6^A switch mechanism and which participate in the processing and maturation of targeted RNA (13). The RNA-binding protein HNRNPA2B1 can bind to the nuclear m^6^A-modified RNA, allowing genes to be spliced (13, 22, 26). IGF2BP1-3 can recognize and bind to m^6^A modification sites, which increases the stability of target RNA and which promotes its translation (10).

## Techniques for Detecting m^6^A Modifications

As early as in the 1970s, m^6^A methylation was identified to modify the mRNA and long non-coding RNA (lncRNA) in eukaryotes (11). However, limited by technical means, the detection especially the quantification of m^6^A and the identification of m^6^A at the single-base level had been progressing slowly (11, 32). The revitalization of researches related to m^6^A modification benefits from the emergence of effective analytical methods. The rapid development of next-generation sequencing (NGS) technologies and the improvement of liquid chromatography sensitivity provide a reliable basis for studying the influence of m^6^A RNA methylation on RNA structure (32–35).

An emerging method called methylated RNA immunoprecipitation sequencing (MeRIP-seq) or m^6^A-seq for identifying m^6^A modification sites on mammalian RNA emerged in 2012, and has recently received widespread attention (34–37). This new method is based on the high specificity of antibodies against m^6^A, and its combination with high-throughput sequencing makes it possible to describe the specific map of m^6^A modification in the mammalian transcriptome. The first step of MeRIP-seq is to fragment the RNA, followed by the use of immuno-magnetic beads with m^6^A antibody to enrich the m^6^A-methylated RNA fragments and the purification of the enriched RNA fragments to construct a high-throughput sequencing library by performing on-machine sequencing. In addition, a common transcriptome library needs to be constructed separately as a control. Finally, the two sequencing libraries are put together for bioinformatics analyses, and the region with a higher degree of m^6^A methylation is obtained, which is also called m^6^A peak (36, 37). The advantage of this method is that it is convenient, fast, and of low cost, and that it can enable a qualitative analysis of the RNA regions that are hyper-methylated. However, MeRIP-seq can only identify the m^6^A sites within RNA fragments of 100-200 nucleotides, and cannot achieve single-base resolution (34). To overcome the low resolution issue, a novel method called m^6^A individual nucleotide resolution crosslinking immunoprecipitation (miCLIP) has marked a major advancement in the field of m^6^A sequencing. This method enhances m^6^A-seq by UV-induced crosslinking of antibodies with immuno-precipitated RNA fragments (35, 38). Other approaches with higher resolutions include site-specific cleavage and radioactive-labelling followed by ligation-assisted extraction and thin-layer chromatography (SCARLET) and photo-crosslinking-assisted m^6^A-sequencing (PA-m^6^A-Seq) (38). Furthermore, it is currently possible to use the CRISPR-based genetic engineering modification methods to directly change any modification site in many organisms and to help develop m^6^A RNA methylation into a research method for more extensive investigations (34).

New breakthroughs have been made in the methods for detecting the overall m^6^A methylation level of cells, including the m^6^A dot-blot and high-performance liquid chromatography-mass spectrometry (HPLC-MS/MS) method to detect the overall m^6^A level of RNA, which can be used to generate important quantitative information on the presence and abundance of m^6^A modifiers (34).

## m^6^A Modification as a Novel Regulator of the Tumor Immune Microenvironment

**Figure 2:**
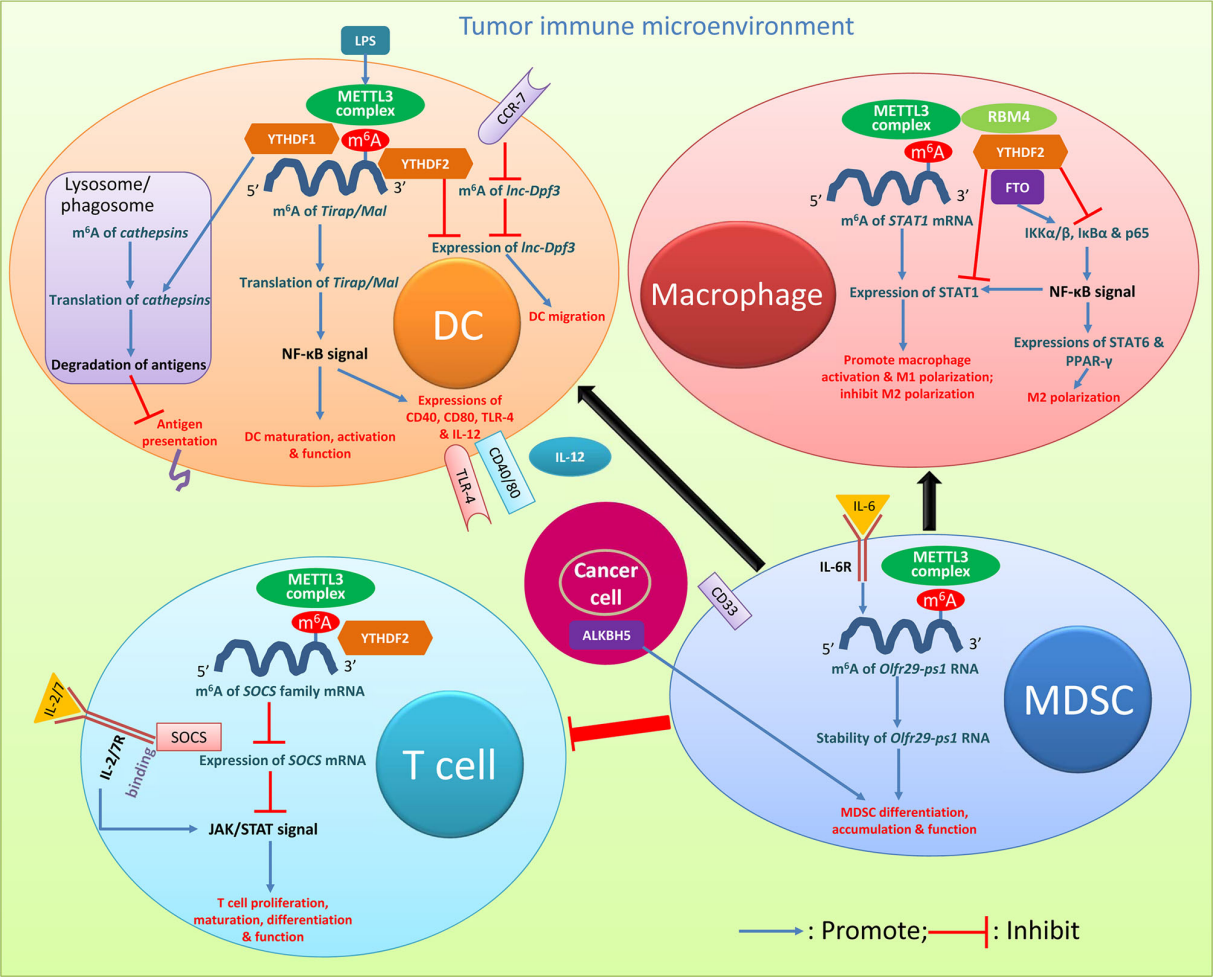
Roles of m^6^A modification in cancer immune regulation. Key m^6^A regulators and relevant pathways and molecules with biology activities clarified in various immune cells are presented. The descriptions are detailed in the relevant texts with citation of this figure. DC, dendritic cell; MDSC, myeloid-derived suppressor cell; LPS, lipopolysaccharide.

Recently, a growing number of studies have emerged mainly focusing on the mechanisms of strengthening anticancer immunity activation. The immune system is divided into innate and adaptive immunity. m^6^A plays critical roles in both the innate and adaptive immune responses and in tumor immunology, which provides important hints for developing various types of antitumor immunotherapies (22, 31). m^6^A also plays a vital role in the complex regulatory network within the TIME, and subsequently affects tumor occurrence, progression, metastasis, and treatment response (39–42). Most of the anticancer immune regulations rely on overcoming the continuous suppression of the adaptive immune response within the TME (43). Immune suppression is a typical feature of the TME, which involves the dysfunction of antigen presenting cells (APCs), recruitment or induction of large numbers of suppressive immune cells, such as CD4+ regulatory T cells (Tregs), dendritic cells (DCs), tumor-associated macrophages (TAMs), and myeloid cell-derived suppressor cells (MDSCs), and secretion of various cytokines (**Figure 2**) (44, 45).

### T and B Cells

T cells regulate the entire adaptive immune response. m^6^A is selectively regulated in tumor infiltrating T cells, and can be an important target in antitumor immunotherapies (44). Regulatory T cells (Tregs) are an important type of T cells which are involved in suppressing inflammation and producing immunosuppression (39). They are a key subset of effector T cells with strong immunosuppressive effects in the TME, and m^6^A-dependent immune function regulation was found in Tregs (44). The family of cytokine signal transduction (SOCS) proteins, mainly including SOCS1-3 and CIS, are inhibitory proteins involved in the transduction of the JAK-STAT signaling pathway, and play a vital role in inhibiting T cell proliferation and differentiation (39, 40, 43–48). The *SOCS* family genes are regulated by m^6^A methylation (44, 49); the modification was induced by the METTL3 complex at the 3’-end of the *SOCS2* transcript, which could be recognized by YTHDF2 (48). Considering the importance of SOCS2 in Tregs in their noteworthy suppressive potential of the differentiation and function of CD4+ T cells and tumor-killing CD8+ T cells in the TME, the roles of m^6^A methylation of *SOCS2* mRNA in T cells in immune response disorders during tumorigenesis should be further explored. In the process of inducing naive T cell development through IL-7 stimulation, the *SOCS* gene controls the IL-7 signaling (46). The SOCS family could act as a mediator binding to IL-7 receptor, which prevents STAT5 activation and inhibits downstream signals involved in T cell maturation and differentiation; T cells are highly responsive to IL-7 signals with downregulation of *SOCS* gene expressions, while upregulation of *SOCS* gene expressions suppresses the IL-7-dependent function of T cells (46). If naive CD4+ T cells are co-cultivated with METTL3-knockout Treg cells, naive T cells will have a greater ability to proliferate due to the complete lack of Treg inhibitory function (11, 50). In CD4+ T cells, reduced m^6^A modification can enhance the stability of the *SOCS* gene mRNA, thereby preventing signal transduction in the IL-2/STAT5 signaling pathway (11, 48, 50). The downregulation of METTL3 makes T cells stay in the naive T cell stage for longer time with reduced METTL3-mediated m^6^A methylation targeting the IL-7/STAT5/SOCS pathway (47). Together, m^6^A modification specifically targets the same class of genes encoding components of essential signaling pathways in different T cell subtypes; m^6^A methylation of *SOCS* mRNA with the involvement of m^6^A modulators including METTL3 and YTHDF2 decreases the stability and expression of *SOCS* mRNA, and reduces the inhibition of SOCS on the JAK/STAT signal, which promotes T cell proliferation, maturation differentiation, and function (**Figure 2**).

Downregulation of the m^6^A writer METTL14 specifically in B cells could result in severe defects in B cell development, with inhibition of IL-7-induced pro-B cell proliferation and blocking of large-to-small pre-B cell transition (51). More studies are needed to further reveal the regulatory roles of m^6^A modification in B cells.

### Dendritic Cells (DCs)

Abundant abnormalities of m^6^A mRNA modifications were found in DCs in cancers. DCs are specialized APCs, which are responsible for the processing and presentation of antigens and the activation of T cell immune responses (11, 39). The regulation of their functions is closely related to the overall balance of immune responses. Exploring the regulatory mechanism of DC function activation is of great significance for in-depth understanding of the inflammatory and immune processes within the TME and for finding potential therapeutic targets for cancers with the involvement of abnormal DC activation (48, 52, 53). DCs have different stages of maturity: Immature DCs induce immune tolerance, mature DCs activate and stimulate immune response, and regulatory DCs downregulate immune response by suppressing T cell response (53). m^6^A methylation mediated by the methyltransferase METTL3 promotes the activation and function of DCs (54). Lipopolysaccharide can induce high expression levels of METTL3 in DCs (55). The specific consumption of METTL3 in DCs leads to impaired phenotype and functional maturation of DCs, and reduces the expression of costimulatory molecules CD40, CD80, and cytokine IL-12 involved in DC maturation. *In vitro* and *in vivo* studies have confirmed that after silencing METTL3 the ability of DCs to stimulate T cell responses is reduced. The METTL3-mediated m^6^A methylation modification of the CD40, CD80, and TLR4 signal transduction junction *Tirap* (also known as *Mal*) transcript enhances the translation in DCs to stimulate T cell activation and enhances the TLR4/NF-κB signal transduction to promote cytokine production; these confirm the new role of METTL3-mediated m^6^A methylation in promoting DC maturation (54). Furthermore, YTHDF1-knockdown mice had stronger response to tumor antigen-specific CD8+ T cells than wild-type mice; knockdown of YTHDF1 in classic DC cells enhanced the cross-presentation of tumor antigens *in vivo* and the cross-activation of CD8+ T cells (30, 40). The transcripts of multiple DC lysosomal *cathepsins* all have m^6^A modification, and YTHDF1 can promote the translation of lysosomal *cathepsin* in DCs by combining these transcripts (30, 56). The depletion of YTHDF1 in DCs attenuated the translation of genes related to the phagosome and lysosomal pathways which are a member of the *cathepsin* family (56). The enzymatic degradation of the proteins in phagosomes after DCs ingestion could destroy antigens to limit cross-presentation of antigens (57–59). The antigen cross-presentation of tumor-infiltrating DCs in YTHDF1-deficient melanoma or colon cancers can induce stronger anticancer immune response, and both *in vitro* and *in vivo*, mature YTHDF1-deficient DCs can induce stronger T cell activation than wild-type cells (57). YTHDF2 is also considered as a potential suppressor of tumor immunity (44). The activation of antitumor T cells and the initiation of anticancer immunity depend on the migration of APCs, especially the migration of DCs to lymph nodes (44, 60). CC-chemokine receptor 7 (CCR7) can stimulate the rapid but transient migration of DCs to draining lymph nodes, *via* upregulating the expression of the lncRNA *lnc-Dpf3* by removing the m^6^A modification to prevent RNA degradation (61). DC-specific *lnc-Dpf3* promotes CCR7-mediated DC migration, leading to excessive adaptive immune responses and inflammatory damage. The m^6^A-dependent manner regulates the dynamic expression of *lnc-Dpf3* in DCs, and YTHDF2 reduces the expression level of *lnc-Dpf3* in resting mature DCs (61). Together, in DCs m^6^A methylation of *Tirap/Mal* mRNA induced majorly by METTL3 strengthened the NF-κB signal, which promotes the expressions of several costimulatory molecules and enhances the maturation, activation, and function of DCs; the m^6^A regulator YTHDF1 promotes the translation of *cathepsins* in lysosomes/phagosomes, which enhances the degradation of antigens thus preventing antigen presentation and the subsequent activation of effector immune cells; YTHDF2 inhibits the expression of *lnc-Dpf3*, and suppresses the CCR-7-induced DC migration (**Figure 2**). The activation of DCs through m^6^A modification has vital roles in promoting the subsequent immune cell activation and function and in the migration of immune cells, thus importantly impacting the initiation and progression of cancers. The breakthrough of researches on the m^6^A epi-transcriptome accelerates the understanding of m^6^A-dependent DC development and activation, serving as prerequisite for future translational exploitation of m^6^A-based immunotherapy. Checkpoint blockade inducing YTHDF1 and/or YTHDF1 depletion in DCs may be a potential immunotherapy strategy (56).

### Macrophages

Macrophages are immune cells derived from the hematopoietic system; they provide important innate immune defenses and maintain tissue-specific functions through regulation of the internal environment within organs (62, 63). For innate immunity, macrophages participate in the regulation of tissue homeostasis and resist viral infection and inflammation (48). Regarding the roles in inflammation and the TME, macrophages can be mainly polarized into the classically activated macrophages (M1 type) with antitumor function and the alternatively activated macrophages (M2 type), the latter of which can inhibit inflammation, promote angiogenesis and tissue repair, and also participate in tumor metastasis (48, 62–67). Upregulation of METTL3 activity greatly promotes the polarization of M1 macrophages, but it has an inhibitory effect on the polarization of M2 macrophages (68). METTL3 directly methylates the mRNA encoding signal transducer and activator of transcription 1 (STAT1), which is the main transcription factor that regulates the polarization into M1 macrophages (57). METTL3-mediated methylation of *STAT1* mRNA significantly increases the mRNA stability and subsequently increases the expression of STAT1. METTL3 may serve as an anti-inflammatory target which drives the polarization of M1 macrophages by directly methylating *STAT1* mRNA (68). Silencing the demethylase FTO significantly inhibits the polarization of M1 and M2 macrophages; FTO knockdown reduces the phosphorylation levels of IKKα/β, IκBα, and p65 in the NF-κB signaling pathway, which in turn leads to the downregulation of STAT1 expression in M1-type macrophages, and of STAT6 and peroxisome proliferation-activated receptor-γ (PPAR-γ) in M2-type macrophages. The actinomycin D experiment showed that silencing FTO could increase the instability of *STAT1* and *PPAR-γ* mRNAs, thereby inhibiting transcription (64). Moreover, when the m^6^A reading protein YTHDF2 is silenced, the mRNA stability and expression of *STAT1* and *PPAR-γ* increase. Silencing FTO can inhibit the NF-κB signaling pathway and reduce the stability of *STAT1* and *PPAR-γ* mRNA through the participation of the YTHDF2 protein, thereby hindering the activation of macrophages (64, 65). FTO contributed to both M1 and M2 macrophage activation. RNA-binding motif 4 (RBM4) interacts with YTHDF2 and can be a possible inhibitor of M1 macrophage polarization *via* the degradation of m^6^A-modified *STAT1* mRNA (69, 70). Together, in macrophages m^6^A methylation of *STAT1* mRNA induced by METTL3 increases the expression of STAT1 and promotes macrophage activation and polarization into the M1 type; the m^6^A eraser FTO can strengthen the NF-κB signal, and promote both the expression of STAT1 for macrophage polarization into M1 type and the expressions of STAT6 and PPAR-γ for polarization into M2 type; YTHDF2 and RBM4 appear to have effects opposite to FTO (**Figure 2**). m^6^A modification majorly impacts the polarization of macrophages thus regulating cancer biology behaviors. These findings may open up new ways to study macrophage polarization and the underlying molecular mechanisms of its involvement in cancers.

### Myeloid-Derived Suppressor Cells (MDSCs)

MDSCs are a group of heterogeneous myeloid cells generally with positive expression of CD33 and with potent immune-inhibitory activity; they have been identified as potential precursors of DCs, macrophages, and granulocytes (71, 72). In the TME MDSCs can suppress immune cells and protect tumors (73). The increase in METTL3 levels in CD33+ MDSCs in the TME is associated with poor prognosis (74). Knocking down METTL3 in CD33+ cells could attenuate MDSC or tumor-related MDSC differentiation *in vitro* (72). In MDSCs the lncRNA pseudogene *Olfr29-ps1* was upregulated by the pro-inflammatory cytokine IL-6; the function of Olfr29-ps1 depended on IL-6-mediated m^6^A modification, and the lncRNA promoted the differentiation and immunosuppressive function of mononuclear MDSCs both *in vitro* and *in vivo* (74). ALKBH5 knockout in tumor cells enhanced the efficacy of immunotherapy, and ALKBH5 could regulate target gene expression and splicing, resulting in changes in metabolite contents and the accumulation of MDSCs (75). Together, METTL3-induced m^6^A methylation of *Olfr29-ps1* which can be stimulated by IL-6 increases the stability of *Olfr29-ps1*, and promotes MDSC differentiation and function (**Figure 2**). The inhibition of MDSCs through the modification of m^6^A methylation may represent a promising anticancer treatment strategy.

## Associations of m^6^A RNA Methylation With Tumor Immunity in Various Cancers

RNA methylation plays an important role in tumor genesis and development. Aberrant RNA methylation has been linked to various human cancers. The expression disorder of m^6^A RNA methylation regulators is closely related to a variety of cancers (48, 76, 77). RNA methylation affects tumor biology by regulating the relevant components of the immune system. It has been found that m^6^A RNA methylation has a variety of biology-regulatory functions during the occurrence and development of cancers *via* modulating tumor immunity (75, 78, 79). The TIME is often characterized by the infiltration of various immunosuppressive cell types, most notably MDSCs and Tregs, and a lack of antitumor immune activity (8, 80). In this section, we summarize the roles of m^6^A modulators in the TIME and immunotherapy within a variety of cancers, and organize the tumor types according to the anatomic systems which the involved organs belong to (nervous system, digestive system, respiratory system, urinary system, reproductive system, hematologic system, and others).

### Nervous System Cancers

#### Glioblastoma and Glioma

In recent years, a large number of studies have proved that the TIME plays a vital role in cancer progression and anticancer therapeutic effects in glioblastoma and glioma (81, 82). Immune cells can penetrate into the brain and form an immune microenvironment. m^6^A regulatory factors are involved in multiple biological processes of tumor progression (50, 83–85). Therefore, clarifying the relationship between m^6^A regulatory factors and TME-infiltrating immune cells can help to assess the anticancer response to immunotherapy in glioblastoma patients (50). 19 m^6^A regulators were highly expressed in glioma tissues (82). The expressions of m^6^A regulatory factors were related to the classification of glioma subtypes. The m^6^A modulators could predict prognosis and therapeutic effects, and were also related to the immune microenvironment of glioma (82, 83). The m^6^A modification regulator ELAVL1 was an effective predictor of PD-L1 treatment efficacy (83). Compared with normal brain tissues and glioblastoma tissues, most m^6^A RNA methylation regulators are differentially expressed in lower-grade gliomas tissues (85). Studies have revealed the correlation between TME infiltration of immune cells and m^6^A modification (86). In glioblastoma, WTAP was found to be overexpressed and to regulate tumor invasion and migration. High expression of WTAP was associated with a low postoperative survival rate. Furthermore, HNRNPC can also impact the invasiveness of glioblastoma cells and is regarded as a potential prognostic biomarker and therapeutic target for glioblastoma (86). Du et al. (87) comprehensively analyzed the m^6^A modification patterns of 1152 low-grade glioma samples, and found that the cases with a low m^6^A score had high immunogenicity and that those with a high m^6^A score were sensitive to chemo-radiotherapy and immunotherapy.

### Digestive System Cancers

#### Gastric and Esophageal Cancers

Zhang et al. (88) reported that m^6^A modification plays an important role in the formation of TIME diversity and complexity by analyzing 21 m^6^A modulators in 1938 gastric cancer (GC) samples. Three m^6^A modification patterns including immune-excluded, immune-inflamed, and immune-desert phenotypes were discovered (88). m^6^A modification patterns could predict the stage of cancer-related inflammation, cancer subtypes, TME matrix activity, genetic variation, and patient prognosis (88–90). The high m^6^A-score subtype had a poor survival rate and had matrix activation with lack of effective immune infiltration; a low m^6^A score was associated with an increased neo-antigen load and an enhanced response to anti-PD-1/L1 immunotherapy (86–89). Assessing the m^6^A modification patterns of individual tumors will help to guide more effective immunotherapy strategies (88). High expression of WTAP was associated with RNA methylation, and its low expression was correlated with strong T cell-related immune responses in GC (86, 89). The infiltration of tumor-associated T cells in the TME was associated with high levels of m^6^A modification, which was mediated by *WTAP* mRNA expression (48, 89). Patients with high WTAP expression had fewer Tregs and CD4+ memory-activated T cells (48, 89). The high infiltration of Tregs and CD4+ memory-activated infiltrating T cells was associated with improved prognosis of GC patients (48, 89). Mo et al. (91) retrospectively analyzed 293 stomach adenocarcinoma samples using data from The Cancer Genome Atlas, and suggested that m^6^A methylation might also be used as an immunotherapy predictor in GCs.

The expressions of m^6^A modulators were correlated with the expressions of immuno-modulators and the level of immune infiltration in esophageal cancer, which can be divided into esophageal adenocarcinoma and esophageal squamous cell carcinoma (ESCC) (92). The m^6^A modulators might improve the responsiveness of ESCC patients to immunotherapy by regulating the TIME and expression of PD-L1 (93).

#### Colorectal Cancer (CRC)

Based on the m^6^A signature score integrating m^6^A-related characteristic genes, patients with colon cancer (CC) could be divided into high- and low-score subgroups; a lower m^6^A score was associated with greater tumor mutation burden, higher PD-L1 expression, and higher SMG (such as *PIK3CA* and *SMAD4*) mutation rates (94, 95). The efficacy of immunotherapy for rectal cancer (RC) is closely related to the level of immune infiltration (96). Low expression of METTL14 in RC led to the downregulation of m^6^A RNA modification, which thereby reduced the level of immune cell infiltration and which led to a poor prognosis (96). The expression level of METTL14 was an independent prognostic factor in RC, and it was positively correlated with the level of immune infiltration. Durable neo-antigen-specific immunity was regulated by m^6^A RNA modification mediated by the m^6^A-binding protein YTHDF1 (56). In a CC-bearing mouse model, YTHDF1-deficient mice showed tumor growth inhibition and survived longer than wild-type mice (56). The loss of YTHDF1 in classical dendritic cells enhanced the cross-presentation of tumor antigens and the cross-priming of CD8+ T cells *in vivo*. In mice receiving anti-PD-L1 immunotherapy for CC, YTHDF1-deficient mice showed a higher cure rate (56). FTO was believed to regulate the methylation of *PD-L1* mRNA thus determining the expression of PD-L1 in CC cells (97). Moreover, in CCs with proficient mismatch repair or low microsatellite instability, deletion of METTL3 and METTL14 increased the infiltration of CD8+ T cells and the levels of IFN-γ, Cxcl9, and Cxcl10 secretion and enhanced anti-PD-1 response (98, 99). CD34/CD276 affected the TIME and was modulated by m^6^A-dependent mechanisms, which ultimately promoted the immune escape of CRCs (100).

#### Hepatocellular Carcinoma (HCC)

Risk stratification by the expressions of 5 m^6^A-related genes (*YTHDF1*, *HNRNPC*, *RBM15*, *METTL3*, and *YTHDF2*) could improve the prognosis prediction of HCC and was related to the response to sorafenib treatment and anti-PD-1 immunotherapy (69, 101, 102). High expression of YTHDF2 was associated with poor prognosis of HCC, and together with increased immune cell infiltration, YTHDF2 might be an independent prognostic biomarker for HCC (103). HCC with low expression of METTL3 had increased dendritic cell infiltration in the TME (98). The levels of m^6^A methylation regulators were related to the overall survival and immunity in HCC, and METTL3, METTL13, YTHDF1, and YTHDF2 might be potential prognosis predictors and therapeutic targets in HCC (104, 105). Du et al. (106) used four m^6^A-related genes to construct a risk feature, which was associated with tumor immunity and which could stratify HCCs. m^6^A regulatory factors were significantly related to the TIME of HCC, which could divide HCCs into two clusters and which were associated with the expression level of programmed death ligand 1 (PD-L1), immune score, immune cell infiltration, and prognosis (107).

#### Pancreatic Cancer (PC)

Tang et al. (108) explored the correlation between M^6^A-related genes and the immune microenvironment of PC, and found that infiltrating immune cells might affect the M^6^A modification in tumor cells. An integrated model called “M^6^AScore” was constructed based on M^6^A modulation factors using RNA-seq data in pancreatic ductal adenocarcinoma-: M^6^AScore-high pancreatic ductal adenocarcinoma was characterized by immune reduction and T cell depletion, and M^6^AScore-low pancreatic ductal adenocarcinoma had higher reaction rates on immune checkpoint inhibitors (ICIs) treatment (109, 110). Thus, the M^6^AScore was associated with the invasiveness and immune status of PC, and could predict PC prognosis and response to ICIs treatment (108, 109, 111). Wang et al. (112) also established a prognostic model based on the expressions of m^6^A regulators, including IGF2BP2/3, KIAA1429, METTL3, EIF3H, and LRPPRC, which was associated with the TIME and immune statuses in PC.

### Respiratory System Cancers

#### Lung Cancer (LC)

Lung adenocarcinoma is the most common histological manifestation of LC and is closely related to m^6^A abnormalities (95, 113–115). m^6^A methylation is reduced in the hyper-immune subtype of lung adenocarcinoma, indicating that m^6^A modification may mediate tumor immunity and provide potential anticancer therapeutic strategies (115). Compared with the low-risk lung squamous cell carcinoma patients, the expressions of ALKBH5, METL3, HNRNPC, and KIAA1429 were significantly reduced in patients with high-risk lung squamous cell carcinoma. It is worth noting that high-risk lung squamous cell carcinoma patients showed more promising treatment responses to PD-1 therapy (115). In non-small-cell lung cancer with high expressions of YTHDF1 and YTHDF2, the densities of four subsets of tumor-infiltrating lymphocytes (TILs; PD-1+, CD8+, Foxp3+, and CD45RO+) were significantly higher (116). High expressions of YTHDF1 and YTHDF2 were related to good prognosis of non-small-cell lung cancer patients, higher TIL density, and downregulation of PD-L1 (116).

### Urinary System Cancers

#### Renal and Bladder Cancers

The expressions of FTO and METTL3 mRNAs were oppositely correlated with the expressions of CD8+ T cell migration-related chemokines in clear cell renal cell carcinoma (ccRCC), which might affect the antitumor immune response (117). Zhong et al. (118) constructed an m^6^A score to accurately evaluate the m^6^A methylation pattern in ccRCC patients, which could be used to predict the anti-PD-1 treatment response in ccRCC. In ccRCC the high m^6^A score group had higher PD-L1 expression, larger numbers of CD8+ T cells and CD4+ FOXP3+ Treg cells, and higher levels of immune cell infiltration (119). Among patients receiving immune checkpoint therapies, the clinical benefits were significantly higher in patients with high m^6^A scores (119). The expressions of 17 m^6^A RNA methylation regulators were closely related to the immunity and malignant progression of papillary renal cell carcinoma (120).

Overexpression of IL-32 is associated with m^6^A modification and good prognosis in bladder cancer, and which may promote the recruitment of CD4+ T cells and dendritic cells, thereby promoting the antitumor effect (121–124).

### Reproductive System Cancers

#### Breast Cancer (BC)

He et al. (125) showed the significant association between RNA methylation levels and the numbers of tumor-infiltrating CD8+ T cells, regulatory T cells, helper T cells, activated NK cells, and M2 macrophages, which indicated the key roles of m6A modulators in the host anti-tumor immune response. Furthermore, the expression pattern of m6A modulators was also significantly related to the expression of PD-L1, TIM3, LAG3, and CCR4, which are well-known T cell depletion targets and important biomarkers in immunotherapy (126, 127) (126). The expression levels of METTL14 and ZC3H13 were significantly positively correlated with the infiltration levels of CD4+ T cells, CD8+ T cells, neutrophils, macrophages, and dendritic cells, and negatively correlated with Treg cells in BC (128). BC genotypes could be divided into two clusters based on four representative m6A regulators (IGF2BP2, IGF2BP3, YTHDC2, and RBM15), which were associated with the number of TILs (129).

#### Ovarian, Endometrial, and Prostate Cancers

The levels of immune cell infiltration and various immune gene markers were closely related to the expressions of RBM15B, ZC3H13, YTHDF1, and IGF2BP1 in ovarian cancer (OC) (130). Gu et al. (131) identified two different m^6^A modes based on 21 m^6^A regulators: A low m^6^A score was associated with immune activation and stronger sensitivity to immune checkpoint inhibitors, while a high m^6^A score was associated with progression of OC.

The expressions of METTL14, ZC3H13, and YTHDC1 were positively correlated with the expression of PD-L1 in endometrial cancer (EC) (132). Knockdown of ZC3H13 or YTHDC1 *in vitro* promoted the malignant phenotype transformation of EC cells.

The expressions of HNRNPA2B1 and METTL3 may also affect the immune microenvironment of prostate cancer (133).

### Hematologic System Cancers

#### Acute Myeloid Leukemia (AML)

Acute myeloid leukemia (AML) is a blood cancer that affects a specific subgroup of hematopoietic stem/progenitor cells, and has different genetic and molecular abnormalities (105, 126, 127, 134). In AML, YTHDF2 could isolate m^6^A-modified circRNA and inhibit innate immunity (125). For unmodified circRNA, it could be used as an effective adjuvant to induce antigen-specific T cell activation, antibody production, and antitumor immunity enhancement (125). The genetic depletion and pharmacological inhibition of FTO significantly inhibited the self-renewal of leukemia stem cells, and induced immune responses by inhibiting the expression of immune checkpoint genes (128). Targeting FTO could make leukemia cells more sensitive to T cell toxicity and overcome immune evasion induced by hypo-methylation agents, suggesting the potential value of targeting FTO in anticancer treatment (128).

### Other Cancers

Knockdown of FTO increased the m^6^A methylation of key tumor-promoting genes in melanoma cells, including *PD-1*, *CXCR4*, and *SOX10*, resulting in increased RNA attenuation mediated by the m^6^A reader YTHDF2 (129, 135, 136). Knockdown of FTO made melanoma cells more sensitive to interferon gamma (IFN-γ), thereby reducing the resistance to anti-PD-1 therapy in mice in an adaptive immunity-dependent manner (129, 137). The combination of FTO inhibition and anti-PD-1 blockade reduced the resistance of melanoma to immunotherapy (86). The expression and mutation statuses of the *ALKBH5* gene were closely related to the response to immunotherapy in patients with melanoma (75). Knockout of *ALKBH5* in tumor cells enhanced the efficacy of immunotherapy, which supported the therapeutic value of ALKBH5 in melanoma immunotherapy (75). Mutation or downregulation of the *ALKBH5* gene in melanoma patients was associated with positive response to PD-1 blockade by pembrolizumab or nivolumab (75).

m^6^A RNA methylation may be involved in the regulation of the immune microenvironment in head and neck squamous cell carcinoma (HNSCC) in synergy with the PI3K/AKT/mTOR signaling pathway (138). Li et al. found that YTHDC2 is associated with the level of immune infiltration of B cells, CD8+ T cells, CD4+ T cells, neutrophils, and dendritic cells in HNSCC (139). Feng et al. (140) further revealed the important role of m^6^A RNA methylation-related lncRNAs in the HNSCC immune microenvironment. Nasopharyngeal carcinoma (NPC) is a highly immunogenic tumor, which is characterized by a large abundance of tumor infiltrating lymphocytes. METTL3 was low expressed in NPC and related to the infiltration of various immune cells (76, 141–144).

Adrenocortical carcinoma (ACC) is a highly immunogenic tumor, and 86.3% of ACCs had abundant tumor infiltrating lymphocytes (145). The m^6^A reader HNRNPA2B1 mediated the pattern of TME infiltration, and promoted the progression of ACC by regulating the activity of macrophages (145).

Numbers of most of the immune cells (type M1 and M2 macrophages, CD8+ T cells, Tregs, and dendritic cells) were negatively associated with IGF2BP2 expression in osteosarcoma (146). M^6^A modification-mediated aberrant activation of cell cycle-related pathways and suppression of immune response may play a crucial role in the progression of osteosarcoma (147).

## Perspectives

m^6^A RNA methylation modification plays various vital roles in nearly all biology processes including cancer initiation and progression. There are some unsolved questions that need to be addressed in the future to fully reveal the function of m^6^A modification during tumor genesis, progression, and antitumor immune response. The roles of m^6^A methylation need to be studied in more types of immune cells and immune-associated cells besides those herein reviewed, and the involvement of m^6^A modification in the regulation of more biology behaviors and functions (e.g., metabolism) of immune cells and in the interplay and crosstalk between cancer cells, immune cells, other stromal cells, and non-cellular TME components need to be further investigated, to comprehensively reveal the complicated m^6^A-associated regulatory networks and to provide promising targets for novel immunotherapy strategies. The m^6^A-modified molecules and the relevant signaling pathways which have been investigated are limited, and the full m^6^A regulatory spectra within specific immune cells need to be further uncovered. Notably, the effects of m^6^A methylation on the biology behavior and function of a certain type of immune cell may vary according to different target RNAs (mRNAs and non-coding RNAs), and the interaction between different regulatory pathways need to be clarified. Besides the extracellular m^6^A inducers (e.g., IL-6 and lipopolysaccharide) herein reviewed, explorations of other inducers and inhibitors may offer better understanding of the meticulous m^6^A regulatory network. The m^6^A regulators which have currently been explored are also limited; deciphering the roles of more m^6^A writers, erasers, and readers and the further interplays between them and identification of the most relevant and potent regulators may provide important hints for development of m^6^A-relevant targets for precision immunotherapy. While the inhibition of YTHDF1 and/or YTHDF2 in DCs and macrophages have been suggested to be promising anticancer strategy, the influences of such inhibition on the other intracellular molecules and pathways besides those already explored and on cancer cells, other immune cells, and other stromal cells also need to be clarified to further support the therapeutic significance with safety; it would also be important to explore how to precisely deliver the corresponding inhibitory drugs to the specific cell types to minimize the off-target effects. It may also be interesting to investigate the impact of m^6^A modification on RNA in different organelles, such as mitochondria and extracellular vesicles. The different regulatory roles of m^6^A methylation in different stages of tumor progression and in primary and metastatic cancer sites and the interplay between m^6^A and other RNA modifications may also be intriguing topics of research.

Most of the currently available evidence on the roles of m^6^A modification in the regulation of tumor immunity in the TME can be divided into two aspects: Exploration of the biology function of a single m^6^A modulator to clarify the underlying mechanism and construction of model or signature integrating multiple m^6^A regulators to precisely predict the prognosis and immunotherapy efficacy within a specific cancer. For mechanism explorations, it is encouraged that the impact of m^6^A modification on more tumor biology behaviors including immune metabolism, extracellular vesicular activity, and autophagy beyond the routinely investigated ones be evaluated. It is important to also look more into the feedback modulation of m^6^A modification by the TIME, and a feedback loop between m^6^A methylation and tumor immunity may better represent the real biology processes within humans. A target RNA is regulated by multiple m^6^A modulators, and a modulator can function on various target RNAs. The interaction between diverse regulatory pathways cannot be neglected, and artificial intelligence (AI) and bioinformatics methods may help to better understand the complex processes. While various m^6^A-based signatures or scores have been constructed, they are mostly based on publicly available databases (e.g., TCGA) which include mostly western populations and often lack validations in different ethnicities. It is warranted that more precision models will be derived from clinically oriented data within different populations and be validated across countries or continents, to largely improve the representativeness and generalizability. The methods used to build the m^6^A-based models are also largely heterogeneous across studies. Comparative studies paralleling investigating the prediction efficacy of models constructed using different methods, including deep learning, other machine learning (e.g., the classical support vector machine), and the popular LASSO regression for selecting more predictively meaningful variables, may help to identify the best model.

Previously, using machine learning, we have constructed two personalized signatures majorly based on multiple selected immune cell markers in the TME which can precisely predict prognosis and chemotherapy benefits for a specific patient (148–150); furthermore, we also build an individualized predictive model based on immune cells in the peripheral blood to help to precisely select the subgroup of patients who will more likely benefit from radical resection (151). It may be interesting to investigate the associations between m^6^A methylation and tumor immunity-based signatures or scores. For m^6^A-based model construction, it is investigable to alternatively change the endpoint from survival outcomes to signatures summarizing characteristics of the TIME, and the models constructed may also be well predictive of longer-term outcomes. Since tissue specimens are not always obtainable especially among patients with advanced or metastatic cancers that are not resectable, it is necessary to fully consider the concept of liquid biopsy and to find surrogate markers within easily accessible samples, especially the peripheral blood. It is encouraged that further studies focus on the association between the m^6^A regulators and tumor immunity markers in the cancer lesion and in easily accessible body liquids to clarify the representativeness of the latter, and then on the clinical usefulness of m^6^A-based models constructed based on the peripheral blood markers. It may also be interesting to assess the efficacy of predicting the previously constructive immune signature and then prognosis and treatment benefits based on scores integrating m^6^A regulators, and models integrating both the m^6^A regulators and tumor immunity components may have further enhanced predictive efficacy.

Based on the close relationship between m^6^A modification and tumor immunity, m^6^A-targeted immunotherapy strategies may be promising. Regulations of the methylation and demethylation statuses within diverse immune cells may dramatically impact the antitumor activities, and rebalancing the homeostasis of m^6^A writers, erasers, and readers may contribute to creating a favorable anticancer immunity effectively inhibiting tumor initiation and progression. m^6^A-based signatures can well predict the efficacy of immunotherapy using agents targeting PD-1/L1, etc., and it is desirable that m^6^A-targeted therapeutic strategies will further enhance the efficacy of immunotherapy; targeting both tumor immunity and m^6^A modification may have markedly stronger effects compared to targeting either alone. It may be recommendable that m^6^A modulators associated with anticancer immune components and activities be selected and form the basis for further drug development; particularly, those with clear and obvious interactions with famous cancer immune markers whose functions have been well revealed (e.g., PD-1/L1, CTLA4, and CD47) should be the focus. Since m^6^A modification is a relatively new concept, there may still be some way to go for the development of relevant agents. Notably, there are complex m^6^A regulatory networks accompanied by numerous feedback loops and other precise regulatory mechanisms within human bodies, and targeting a single modulator may not fully produce the anticipated effects. Action on multiple m^6^A modulators to reshape the relevant homeostasis may have better anticancer effects. Furthermore, to minimize the possible off-target effects, it may be desirable to more precisely deliver the m^6^A-targeted modulatory agents into the immune cells of interest, with the help of some advanced drug delivery technologies (e.g., nanotechnology). Importantly, it is unclear whether change in level of a specific modulator will cause a dramatic cascade reaction greatly impairing other body functioning, and the influences of the “butterfly flapping its wings” should be carefully assessed before any m^6^A-targeted agents are used in humans.

## Conclusions

This review focuses on the modulation of immune function by m^6^A in the TME of various cancers. m^6^A is one of the most common RNA modifications and affects tumor occurrence, development, and response to immunotherapy by regulating anticancer immunity. The m^6^A writers, erasers, and readers, which are involved in nearly all biology processes of RNA, such as maturation, transport, splicing, translation, and degradation, all play important roles in the modulation of anticancer immune response. m^6^A can impact anticancer immunity by regulating diverse activities of various immune cells, such as the differentiation of T cells, the stabilization of Tregs, the maturation of DCs, the polarization of macrophages, and the function modulation of MDSCs. m^6^A modulators are closely related to tumor immunity and immunotherapy, and many abnormally expressed m^6^A regulatory factors can impact anticancer immune functions and further modulate tumor genesis, proliferation, growth, invasion, and metastasis by regulating the balance between the expressions of oncogenes and tumor-suppressor genes in the TIME. Notably, most of the reviewed studies on the relationship between m^6^A RNA methylation and tumor immunity are still in their infancy, and more in-depth researches are needed to explore the mechanisms underlying the regulation of tumor immunity by m^6^A modifications.

## Author Contributions

Conception or design: LG, YS, and LH. Literature retrieval and review: LG and LH. Drafting of the manuscript: LG and LH. Critical revision of the manuscript for important intellectual content: HY, CZ, YS, LH, and JZ. Administrative, technical, or material support: YS and JZ. All authors contributed to the article and approved the submitted version.

## Funding

This study was sponsored by Shanghai Pujiang Program (21PJ1409700) and by the Start-up Fund for the Introduction of High Level Talents by Ruijin Hospital, Shanghai Jiao Tong University School of Medicine. The funders had no involvement in study design; in the collection, analysis, or interpretation of data; in the writing of the report; or in the decision to submit the paper for publication.

## Conflict of Interest

The authors declare that the research was conducted in the absence of any commercial or financial relationships that could be construed as a potential conflict of interest.

## Publisher’s Note

All claims expressed in this article are solely those of the authors and do not necessarily represent those of their affiliated organizations, or those of the publisher, the editors and the reviewers. Any product that may be evaluated in this article, or claim that may be made by its manufacturer, is not guaranteed or endorsed by the publisher.
